# Remarks on the Use of the Terms “Congestion” and “Congestive”

**Published:** 1847-02

**Authors:** Thomas C. Brown

**Affiliations:** Woodville, Miss.


*Remarks on the use of the terms "Congestion" and "Congestive."* By THOMAS C. BROWN, M. D., of Woodville, Miss.—The term *Congestive Fever* has become so familiar in the South and West, that the young and inexperienced are led to look for a distinctly marked and peculiar fever, and are sadly perplexed in their investigations. At one time it is described as "algid intermittent fever," at another as "malignant or pernicious remittent fever." They find it supervening on intermittent, remittent and continued fevers—on gastritis and gastro-enteritis—on typhoid pneumonia—on epidemic dysentery; and in fact, as complicated with most of our summer and autumnal diseases, and sometimes with our winter epidemics.

Would it not be well for the medical profession, if our journalists and teachers of medicine, would teach that "there is not, properly, any fever characterized by *congestion* in contrast with another which is without it;" but that *congestion* is common to nearly all fevers, whether intermittent, remittent, or continued; and that it is not limited to any one organ; the brain, the spinal column, the lungs, the liver, the spleen, the kidnies and abdominal viscera, may be the seats of congestion, either separately or several of them at the same time; and these as they are separately or severally affected, will characterize the symptoms according to the organ or the several organs which may be involved?

The term *Congestion* comes from the Latin *congero*, to amass, and in a medical sense conveys the idea of turgescence or fulness of blood in a particular part, and implies simple engorgement and over distension of the blood vessels. It seems rather the result of irritation than of inflammatory action, and when it passes into inflammation, it would seem to be more dependent on the general state of the constitution, on the plethoric condition of the blood vessels, with the tendency towards inflammatory excitement, than on the primary irritation which occasioned it. The presence of internal congestions must, of course, be inferred from the symptoms which indicate them, and to connect the symptoms with the structural lesions which they denote, so as to furnish practical guidance in the treatment of them, is the proper object of medical research.

Their treatment must depend in part on the nature of their site, but principally on the cause which occasions them. If congestion result from a plethoric habit, mere local treatment can never give permanent and effectual relief; on the contrary, if the congestion be owing to irritation only, then general depletion is unnecessary, and would be injurious.

Congestion generally precedes, but may follow inflammation, by the irritation set up in neighbouring organs, or be transmitted to those more distant by nervous influence. Strong innervation, in which the nervous centres are much excited as under various emotions, will produce congestion, at one time of the brain, at another of the lungs or liver. Certain nervous affections, as hysteria, will give rise to irregular determination and retardation of blood, producing congestion in some organ, which often entirely disappears after the removal of the nervous disturbance. Congestion dependent on mere nervous disorder, will generally be irregular or periodical in its appearance, but if there be a permanent disorder of function in an organ, the congestion depends upon permanent irritation. In the commencement of fevers the congestion is of an irregular or periodic character, but when occurring in the latter stages of fever, it is usually of a more permanent and dangerous character.

Congestion, coming on in the progress of intermittent and remittent fevers, is of frequent occurrence, and when supervening in the latter stages of these diseases, it generally indicates great danger; but when it ushers in an intermittent fever, and the paroxysm is followed by very feeble reaction, it indicates great danger.

The symptoms of the congestion of a particular organ, or of several organs, combining with the sympathy of a particular fever or disease, upon which it supervenes, must, necessarily, give rise to great diversity of symptoms, and will require a variety of treatment as dictated by the existing circumstances. Hence the confusion which results in an attempt to study the symptoms of congestive fever, as a distinct species of fever. If we are to have a *nosology*, in which *congestion* is to be the cognomen of a particular species, we must adopt a division of fever somewhat like the arrangement of Armstrong, and dispense with the distinctions of fever into intermittent, remittent and continued.—*New Orleans Med. and Surg. Journ.*

